# A Real-Time Vehicle Detection System under Various Bad Weather Conditions Based on a Deep Learning Model without Retraining

**DOI:** 10.3390/s20205731

**Published:** 2020-10-09

**Authors:** Xiu-Zhi Chen, Chieh-Min Chang, Chao-Wei Yu, Yen-Lin Chen

**Affiliations:** Department of Computer Science and Information Engineering, National Taipei University of Technology, 1, Sec. 3, Chung-hsiao E. Rd., Taipei 10608, Taiwan; t107599006@ntut.edu.tw (X.-Z.C.); t107598023@ntut.org.tw (C.-M.C.); david741002@ntut.edu.tw (C.-W.Y.)

**Keywords:** intelligent traffic system, vehicle detection system, various bad weather conditions

## Abstract

Numerous vehicle detection methods have been proposed to obtain trustworthy traffic data for the development of intelligent traffic systems. Most of these methods perform sufficiently well under common scenarios, such as sunny or cloudy days; however, the detection accuracy drastically decreases under various bad weather conditions, such as rainy days or days with glare, which normally happens during sunset. This study proposes a vehicle detection system with a visibility complementation module that improves detection accuracy under various bad weather conditions. Furthermore, the proposed system can be implemented without retraining the deep learning models for object detection under different weather conditions. The complementation of the visibility was obtained through the use of a dark channel prior and a convolutional encoder–decoder deep learning network with dual residual blocks to resolve different effects from different bad weather conditions. We validated our system on multiple surveillance videos by detecting vehicles with the You Only Look Once (YOLOv3) deep learning model and demonstrated that the computational time of our system could reach 30 fps on average; moreover, the accuracy increased not only by nearly 5% under low-contrast scene conditions but also 50% under rainy scene conditions. The results of our demonstrations indicate that our approach is able to detect vehicles under various bad weather conditions without the need to retrain a new model.

## 1. Introduction

Numerous vehicle detection methods have been proposed to obtain trustworthy traffic data for the development of intelligent traffic systems. Briefly, we can split these methods into two categories: the first category adopts the classical machine learning models trained from manually defined features [1,2,3,4,5,6], such as Haar-like features and histogram of gradients (HoG) features with AdaBoost classifiers and support vector machines (SVM) classifiers; the other category comprises deep learning model-based approaches constructed from large amounts of labeling data [7,8,9,10,11]. Both categories can currently satisfy real-time requirements, yet the detection and classification accuracies of deep learning model-based approaches are still more robust than machine learning models constructed from manually defined features. Compared to the machine learning techniques in earlier decades, deep learning is more powerful due to its detection ability in abstraction.

Composed of multiple layers, the deep learning structure is able to extract potential features from training data and develop a more robust strategy, such as object detection tasks; it obtains higher detection rates than traditional machine learning models. Region-based Convolutional Neural Networks(R-CNN) [12] is one successful application of deep learning for object detection and classification tasks; it is comprised of a region proposal, feature extractor and classifier. The region proposal was accomplished through selective searching [13,14], the feature was extracted by AlexNet deep convolutional neural network (CNN) and the linear support vector machine was trained. To reduce the detection time, a spatial pyramid pooling network and region of interest pooling layer (RoI pooling) were adopted; then, the linear SVM was replaced by a fully connected layer, resulting in a new model named Fast R-CNN [15]. For further improvement, Faster R-CNN [16] completed the region proposal task through a new concept module, the Region Proposal Network (RPN), that functions as an attention mechanism. Although the new concept efficiently improved the computational time, it still only reaches a rate of 17 fps on a desktop computer platform, which is not sufficient for real-time applications.

**Figure 1:**
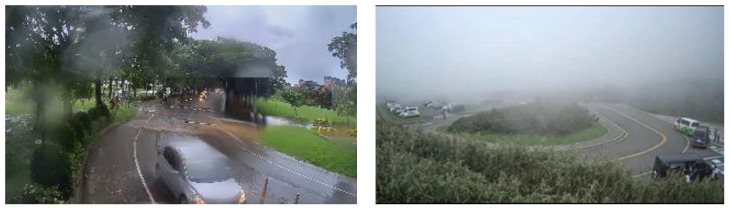
Extreme weather conditions.

YOLO (You Only Look Once) [17], a well-known model structure for its high-speed inference that is able to complete object detection and classification in real-time, has been published. The model first splits the input image into grid cells in which each cell is responsible for predicting the object of the one whose bounding box center is located in it. Following its main concept, the research team of YOLO adopted different technique concepts from other existing models, such as pre-defined anchor boxes, batch normalization, feature pyramid network, etc., and developed the second [18] and third versions of the model [19]. With an input size of 320 × 320, YOLOv3 [19] is able to complete the inference in 22 ms at 28.2 mAP, which is three times faster than Single Shot MultiBox Detector (SSD) [20] with the equivalent accuracy. Under common scenarios, such as sunny or cloudy days, most of the above-mentioned deep learning-based detection methods can perform sufficiently well; however, the accuracy drastically decreases under various bad weather conditions such as rainy days or days with glare, which normally happens during sunsets, as shown in Figure 1. This issue may cause an intelligent traffic system to obtain incorrect data and make incorrect decisions that lead to serious problems, such as traffic accidents, improper control of traffic lights, etc. Smart traffic has become a modern trend, the development of which countries pay a great deal of attention to; however, under the influence of climate change, most countries are suffering from extreme weather conditions. For this reason, a detection system which has high accuracy is needed worldwide.

As for the issue of vehicle detection under various bad weather conditions, there are a large number of existing research studies about image restoration for object detection. Li et al. [21] proposed a method that imposes priors, which are based on Gaussian mixture model learning, for background and rain streak layers. The main idea of this method is to decompose the rain streak from the captured image and obtain the recovered background. Luo et al. [22] introduced the shape prior technique to avoid the incorrect decomposition of the rain streak layer. Gary and Nayar [23] observed that the brightnesses of raindrops are approximately similar regardless of the background intensity. Based on this phenomenon, the candidate raindrops were detected and filtered by predefined constraints. After computing the spatio-temporal correlation between the previous and following frames and determining the pixel locations of raindrops by the directions of correlation, the target pixels are restored by the interpolation of the pixel value from the previous and following frame. Shen and Xue [24] also located the positions of raindrops through intensity variation but restored the pixel value not only on the target pixel but also its neighborhood and obtained a greater smoothing result. Although the existing methods obtain satisfactory results in terms of removing the rain stripes, raindrops on lenses still seriously affect the vehicle appearance on the image frame. Furthermore, for a real-time system, only past frames can be taken as the reference frames; as a result, the restoration methods are not suitable for our application. Another approach for this issue is to collect specific data to retrain the deep learning model. Leung et al. [25] collected nighttime images by themselves and labeled all of them to develop a nighttime vehicle dataset. With the dataset, they trained the neural network and obtained a model that achieved the purpose of detecting vehicles with multiple classes. To deal with the nighttime scenario, the creation of a dataset for a specific purpose may work; however, there are many different extreme weather conditions, and building datasets for each of the cases does not tackle the problem at its roots. In addition, the hardware requirement of training a new deep learning model is much higher than just taking deep learning model as inference tasks. Further, training a deep learning model for all possible bad weather conditions suffers from huge computational times to converge the corresponding model weights and parameters; therefore, a visibility complementation module that can be combined with the exist vehicle detection method and can improve the detection accuracy for different weather conditions without retraining the classification model or deep learning model is needed.

To resolve the above-mentioned issues of vehicle detection under various bad weather conditions without the cost of completely retraining the deep learning models for different weather conditions, this paper proposes a new vehicle detection system with a visibility complementation module that is integrated with multiple deep learning techniques. The complementary module assesses the visibility and adopts different visibility correction methods depending on the assessment result. The module improves the detection accuracy under various bad weather conditions based on the unrestrained deep learning model. The considered parameters were not specific for local applications, such as the image contrast value and V value in the Hue-Saturation-Value (HSV) color space; as a result, the proposed method can be adapted in various traffic scenes and environments in different countries. We validated our system on multiple surveillance videos of different various bad weather conditions and proved that our system is able to detect vehicles under various bad weather conditions without retraining a new model; in other words, the proposed system overcomes the difficulty of detecting vehicles under various bad weather conditions through a pretrained deep learning model. 

The remainder of this paper is organized as follows. In Section 2, we present details of the proposed vehicle detection system, including the vehicle detection module and visibility complementation module. In Section 3, we present the experimental and comparative results with both visualization and numerical examples. In addition, the computational time is also analyzed to demonstrate the real-time performance. Finally, the conclusion is given in Section 4.

## 2. The Proposed Method

**Figure 2:**
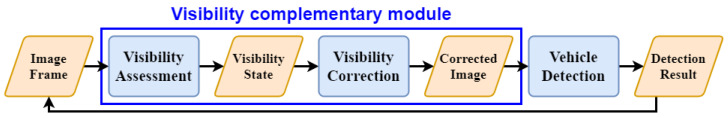
System flowchart.

The proposed system includes a visibility complementation module and vehicle detection module, as shown in the flow chart in Figure 2. The visibility complementation module is responsible for assessing the visibility of the current image frame and corrects the image frame through different deep learning-based approaches depending on the assessment results. The vehicle detection module is purely responsible for obtaining the vehicle position and the bounding boxes in the current image frame implemented based on a YOLOv3 [19] structure. The acronyms of the parameters used in the proposed method are shown in Table 1. The details of the two modules are described below.

### 2.1. Visibility Complementation Module

This module comprises an assessment unit and a correction unit. By observation, the most common reasons for the accuracy to drastically decrease are that vehicles are covered by haze and glare and that raindrops adhere to the camera’s lens; therefore, the assessment unit evaluates the input image frame to determine the quality state and locate the adherent raindrop locations, forming a visibility state result. Depending on the visibility state result, the input image frame will be corrected through different visibility correction approaches. The details of the two main parts are described below.

#### 2.1.1. Visibility Assessment

**Figure 3:**
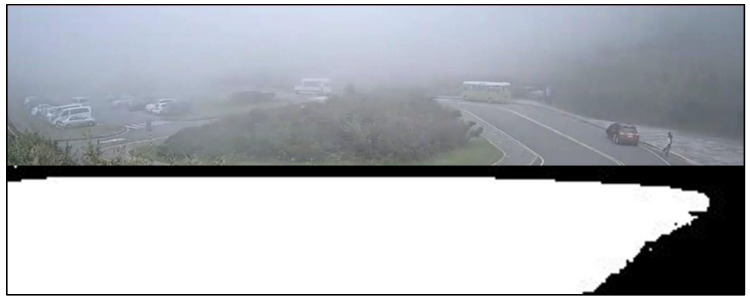
Contrast property assessment result.

**Figure 4:**
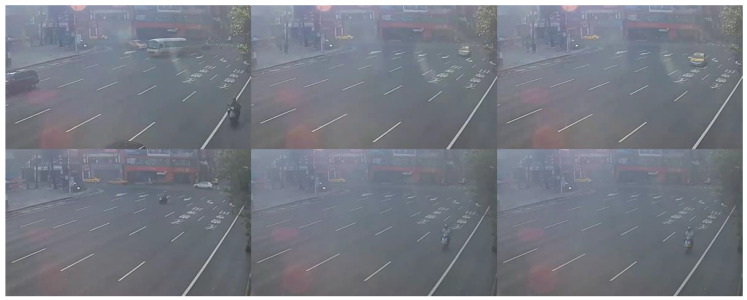
Collected sample images of the partitions affected by sunset.

With regard to the quality state, we are concerned with the contrast and the amount of the pixels with high values in the entire image frame. If one of the statements is satisfied, the current image frame will be determined as a low-contrast image frame. To obtain the contrast property, we adopted the Laplace operator. The Laplace operator takes not only the specific direction but all of the neighboring pixels’ gradients into account. By applying the Laplace operator to the whole image frame pixel by pixel, we can obtain the resultant contrast image, $Image_{c}$; then, the standard deviation SD of the whole image frame will be calculated with Equation (1):

(1) $$\mathrm{SD}\left(Image_{c}\right)=E{\left[{\left(Image_{c}-\mu_{c}\right)}^{2}\right]}^{1/2}$$

where E() indicates the mean operation and $\mu_{c}$ represents the mean value of $Image_{c}$. If the standard deviation is located in the defined threshold region $T_{c}$, the visibility of the image frame may be affected by haze. The defined threshold region $T_{c}$ in our experiment was set as [95, 110]; the sample result of the contrast assessment is shown in Figure 3. To confirm whether the glare from the sunset affects the visibility or not, in this study, we directly counted the number of pixels in which value in the HSV color space was larger than the defined threshold $T_{v}$. This threshold value was determined based on our experiments with 3265 collected partitions affected by sunset, and the resultant average values were all about 120. Thus, the threshold $T_{v}$ was set as 120 to obtain satisfactory results. The sample images of the partitions affected by sunset are shown as Figure 4. If the ratio of the pixels with high values to the whole image frame pixels $R_{high\_v}$ reached the defined ratio threshold $T_{high\_v}$, the image frame may have been affected by glare caused by sunset. In order to reach real-time requirements, the defined ratio threshold $T_{high\_v}$ in our experiment was set as 80%, which means that, unless almost the whole image frame is covered by glare, effects due to glare will not be corrected. The low-contrast image frame decision formula is defined in Equation (2).

(2) $$\mathrm{Low}\;\mathrm{contrast}=\left(T_{c}{}_{lower\_bound}<\mathrm{SD}\left(Image_{c}\right)<T_{c}{}_{upper\_bound}\right)\;||\;(R_{high\_v}>T_{high\_v})$$

**Figure 5:**
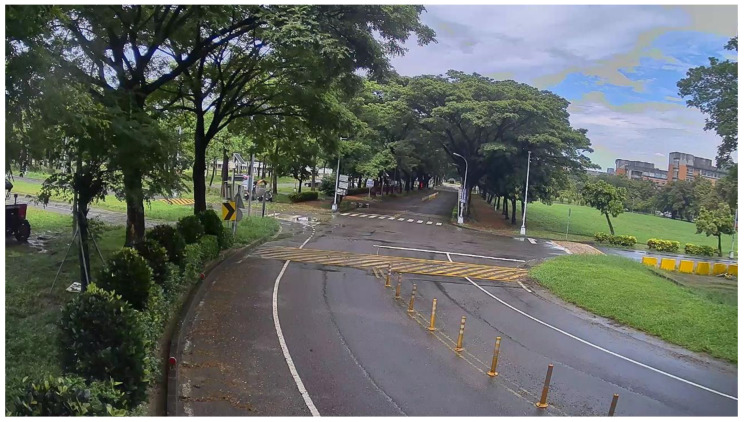
Sample image of the scene base frame.

**Figure 6:**
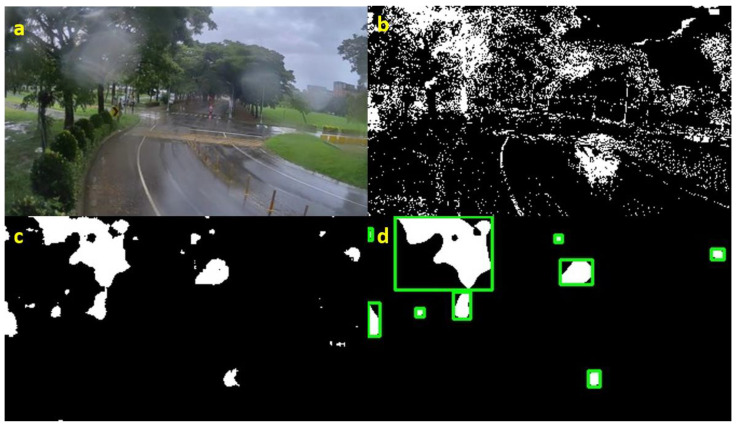
Processing flow for locating adherent raindrops. (**a**) Input image frame with adherent raindrops. (**b**) Difference result between the current frame and scene base frame on the value plane under the HSV color space. (**c**) Denoising result through medium blur. (**d**) Connected component visualization result.

To locate the adherent raindrops, a scene base frame and several conditions are needed. The scene-based frame can be selected from any frame that is clear enough to recognize the objects in the scene, as shown in Figure 5. First, from observation, the partitions affected by adherent raindrops cause an increase in brightness, even becoming a bright spot on image frame; therefore, we obtained the proposal raindrop position through the difference between the current frame and scene base frame on the value plane in the HSV color space; the resulting image is shown in Figure 6b. Next, a medium blur operation was adopted for denoising so that the result could be more reliable; the result of this is shown in Figure 6c. Lastly, the numbers and locations of the prior raindrops were observed by applying a connecting component algorithm to the denoised image frame; the visualization result is shown in Figure 6d. Irrespective of the kind of scene, several scenarios that cause a variance of pixel values exist; as a result, a threshold $T_{component}$ for the number of components was set. Due to haze and fog appearing as a large connected area of pixel value variance, the number of the components calculated through the above steps was usually not very large; as a result, if the number of the prior raindrops was more than the defined threshold $T_{component}$, it was confirmed that some adherent raindrops existed on the camera lens. The threshold for $T_{component}$ was set as 3 in our experiment.

**Figure 7:**
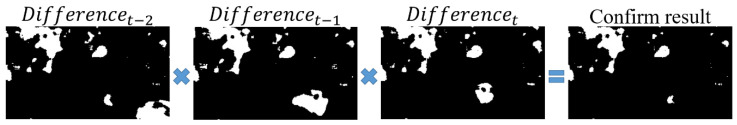
Concept of the confirmation strategy.

As we know, there are a large number of noises that might affect our location results, such as flashlights, moving trees and leaves, etc. To overcome this issue, we proposed a confirmation strategy that made our location result more robust. Based on the location results of each input frame, the confirmation strategy checked for the consistency against the previous K−1 image frames, where K is an integer that represents how many frames will be confirmed together; unless K frames in the same position were all determined to show the presence of raindrops, the position was determined as a clear state pixel. Adherent raindrops will exist on the image frame for a long time, while others will not; consequently, this method is efficient for locating the raindrops in a more accurate way. Figure 7 shows the result of assuming K=3 for confirmation.

To ensure that the conditions and parameters in this research were widely supported, the proposed method was evaluated on multiple surveillance videos collected from roadside traffic surveillance systems set up in Kaohsiung, Keeling and Yangmingshan National Park in Taiwan. The analysis results are shown in Table 2. For this analysis experiment, we collected four scenes under different weather conditions, including glare, haze, sunny and rainy conditions. By applying the statement defined in the proposed method, the processed results demonstrated that the adopted parameters were able to obtain efficient results in various weather conditions.

#### 2.1.2. Visibility Correction

After receiving the visibility state result, the corresponding correction methods could be adopted. If the current image frame was noted as being low contrast, it was corrected through a dark channel prior [26]; to accelerate the correction processing, parallel processing was applied. The partitions affected by adherent raindrops were restored with deep learning restoration techniques. The details are described as below.

Image frames with haze or glare cover can be represented with the following equation:

(3) I(x)=J(x)t(x)+A(1−t(x)),

where ***I*** is the captured image frame, ***J*** is the scene information without noise, ***A*** is the global atmospheric light and *t* is the transmission from scene to camera. Fattal [26] proposed a scene information restoration method based on the formula above through Independent Component Analysis (ICA). Although the proposed method obtains good results, it might obtain unreliable results sometimes because of the non-satisfied statistically independent assumptions. The dark channel prior [26] estimates the transmission directly and improves the restoration reliability.

He et al. [26] observed that at least one of the color channels exists in most of the non-sky patches from the image frame, and the color channel has a very low intensity patch. This discovery can be represented with the formula below:

(4) $$J^{dark}\left(x\right)=min_{c\in \left\{r,g,b\right\}}\left(min_{y\in \Omega \left(x\right)}\left(J^{c}\left(y\right)\right)\right),$$

where $J^{c}$ is a color channel of J and Ω(x) is a local patch centered at x. $J^{dark}$ is the dark channel of J, which is close to zero. After making some derivation of applying dark channel prior into the scene image formula between sky and non-sky regions, a robust haze removal method was proposed.

**Figure 8:**
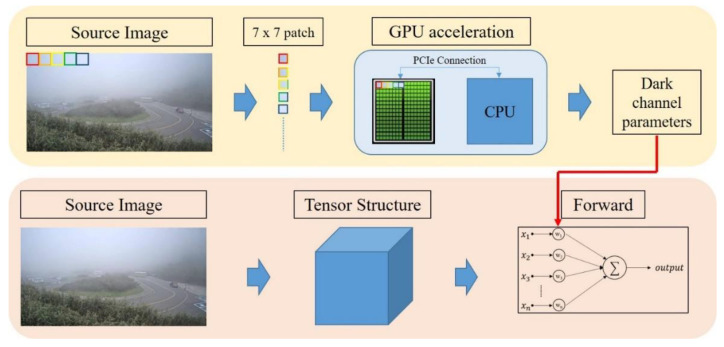
Graphics processing unit (GPU) acceleration flowchart.

**Figure 9:**
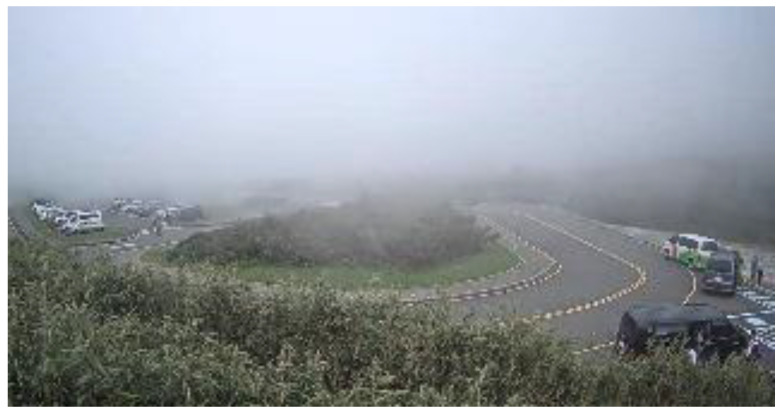
Sample image of haze scene.

**Figure 10:**
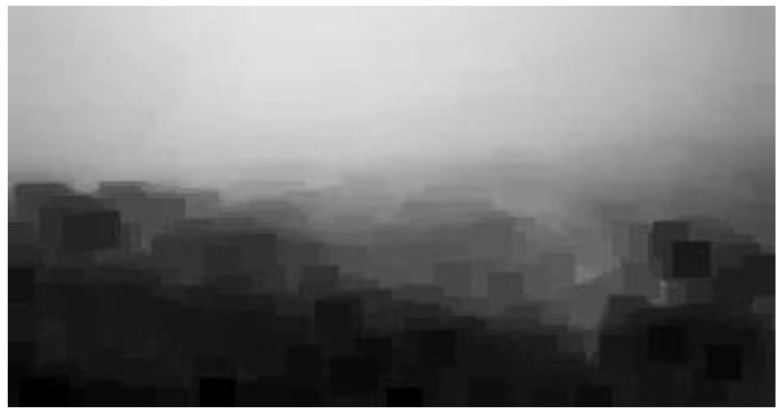
Low-intensity visualization result.

For our experiment, a 7×7 patch size was the most suitable setting for searching for the lowest intensity value in the target patch between RGB color channels. To satisfy the real-time requirements, we assigned haze removal computations to a graphics processing unit (GPU) and shortened the processing time. The GPU acceleration was implemented through PyTorch 1.4.0 [27], an open source machine-learning library based on the Torch library. We loaded the image patches as a tensor structure and obtained its minimum intensity by using PyTorch Application Programming Interface (API). After calculating the dark channel parameters, all source images were applied to the formula above; after calculating parallels with the GPU, we finally obtained the de-hazed image frames. The whole processing flowchart is shown in Figure 8. Figure 9 presents a sample image frame of a low-contrast state, and Figure 10 illustrates the visualization result of dark channels. In this figure, we can clearly recognize that the partitions affected by haze were correctly detected.

**Figure 11:**
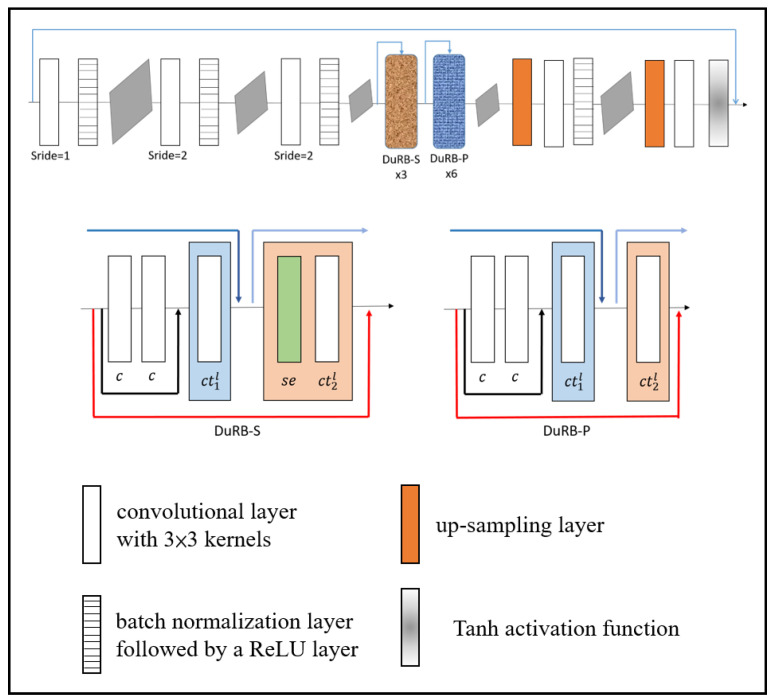
Raindrop removal network structure.

**Figure 12:**
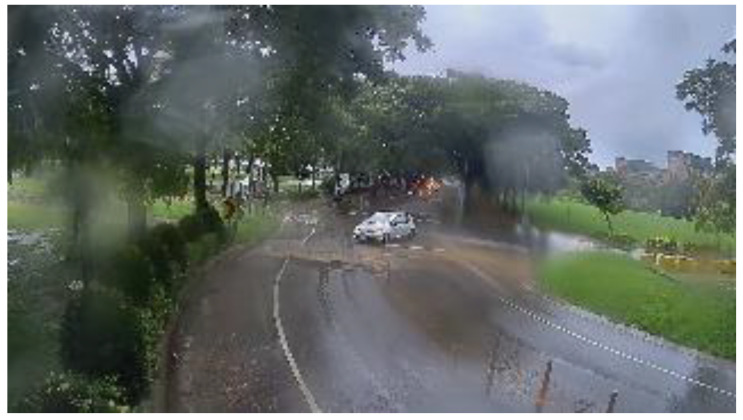
Sample image of a rainy day scene.

**Figure 13:**
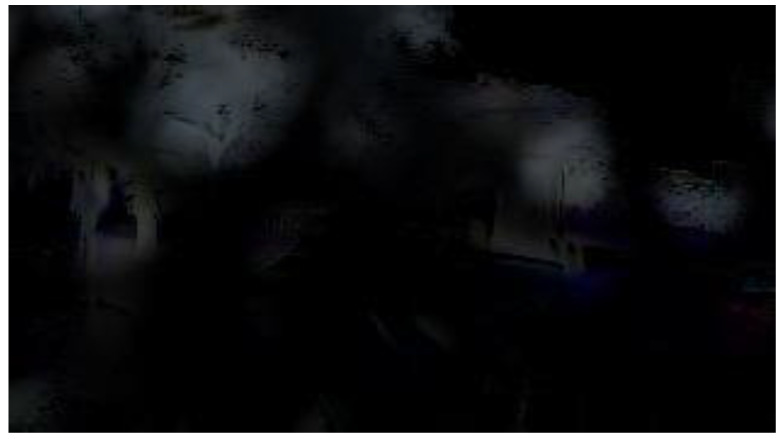
Attention map of the rainy day scene sample image.

The partitions affected by adherent raindrops were restored through a dual residual network. Liu et al. [28] proposed an image restoration method based on dual residual networks which can be applied to noise removal, motion blur removal, haze removal, raindrop removal and rain-streak removal. For rainy day scenes, the main negative effects result from raindrops; therefore, we adopted the raindrop removal method in our proposed system. The raindrop removal network structure is a convolutional encoder–decoder network with dual residual blocks, resulting in a Dual Residual Network-S-P (DuRN-S-P) structure, as shown in Figure 11. The attention mechanism generated an attention map of the input image frame, which showed the raindrops’ locations. Focusing on the raindrops’ locations and restoring them, we could finally obtain the restoration image frame and were ready to detect the vehicles in the frame. Figure 12 presents the sample image of a rainy day scene, and Figure 13 shows the attention map, which is the visualization result of the attention mechanism. 

### 2.2. Vehicle Detection Module

**Figure 14:**
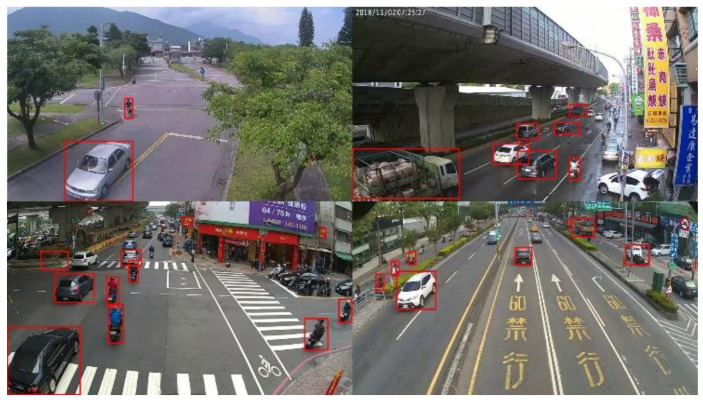
Sample images from our self-collected dataset.

**Figure 15:**
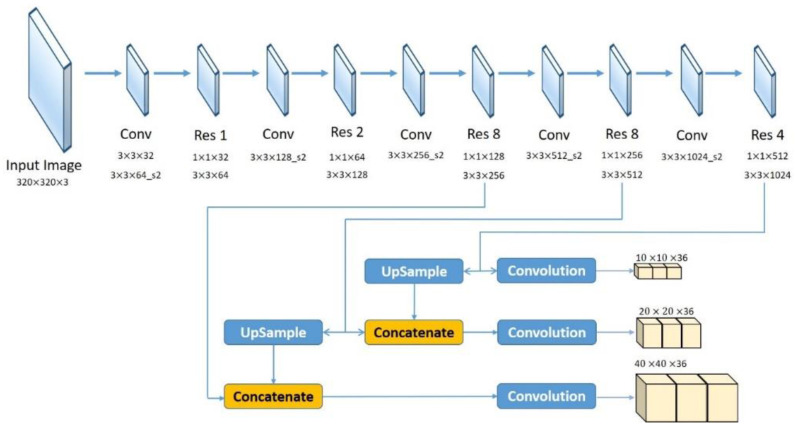
You Only Look Once (YOLOv3) [19] network structure.

To accomplish the real-time requirements and obtain accurate detection results under large-scale conditions, this study adopted YOLOv3 [19] as a detection method. YOLOv3 [19] uses Darknet-53 as its backbone, and the residual blocks in it can deal with vanishing and exploding gradient problems. To improve the capability of detecting small vehicle objects, the Feature Pyramid Network (FPN) was applied in the network structure. Through FPN, the network combined the information of global features in the shallow part and local features in the deep part of feature maps and developed a feature map with less information loss that made the objects easier to detect. Due to the different view-points of the image between public datasets and our application scenario, we collected the vehicle image dataset by ourselves in this study. Our collected datasets contained seven types of vehicles, including sedans, motorcycles, pedestrians, bicycles, trucks, buses and special purpose vehicles, and a total of over 100,000 labeled instances in 34,090 images with 1280 × 720 resolutions were collected. A sample image of our self-collected dataset is shown in Figure 14. After setting the network input image size to 416 × 416 and modifying the output dimension of each detection scale, the YOLOv3 [19] structure of our proposed method was as shown in Figure 15.

## 3. Experimental Results

In this section, the visualization results of the visibility correction and detection improvement under three different bad weather conditions are shown, and the comparative numerical results between adopting the proposed method or not are also calculated. The experimental platform of the proposed system was implemented on a Ubuntu 16.04.4 LTS (Xenial Xerus, Canonical Ltd., London, UK) 64 bit operating system with Python 3.5. The hardware environment was as follows: Intel^®^ Core™ i7-7700K, Double Data Rate Fourth Generation(DDR4) 32G and a GeForce GTX 1070 Ti GPU.

### 3.1. Visualization Results

**Figure 16:**
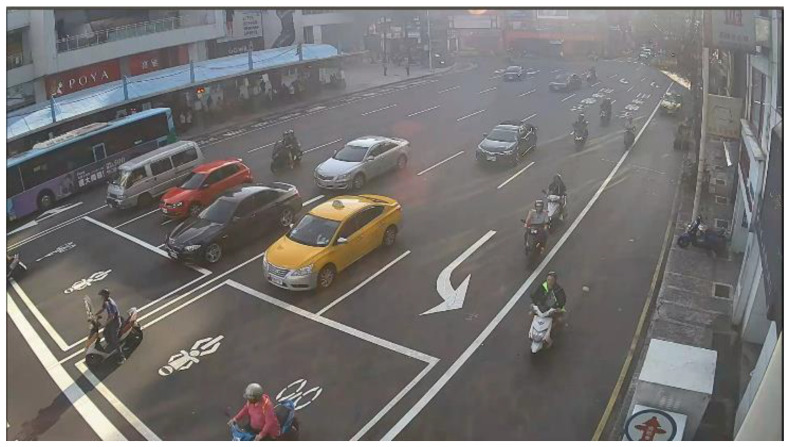
Glare scene: original image.

**Figure 17:**
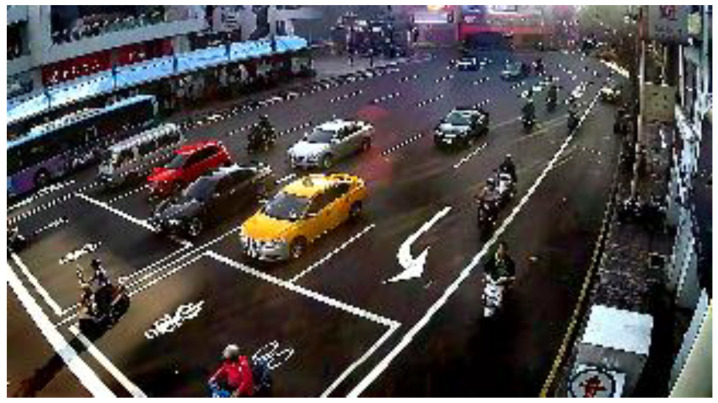
Glare scene: correction result.

**Figure 18:**
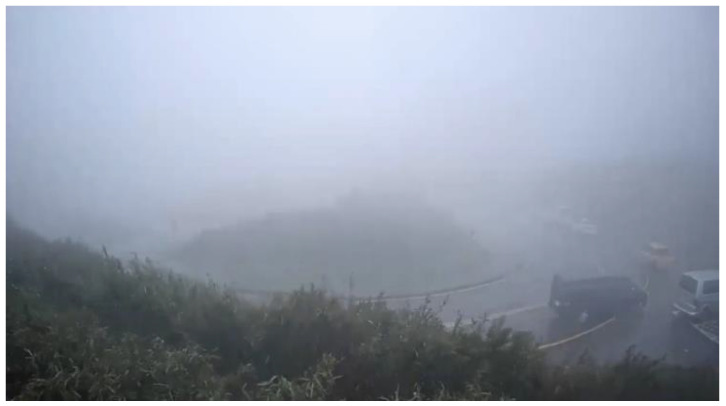
Haze scene: original image.

**Figure 19:**
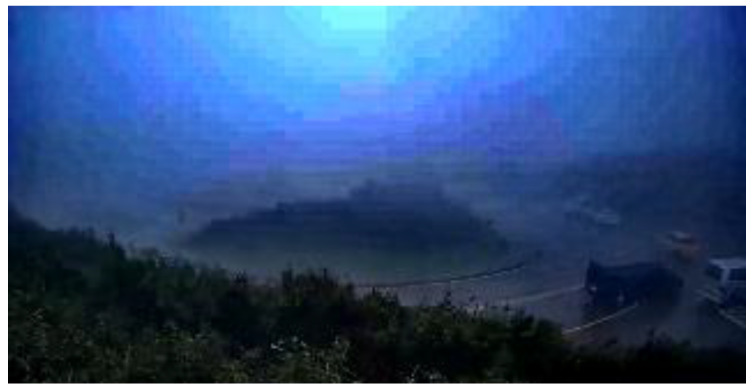
Haze scene: correction result.

We cooperated with the Department of Intelligent Systems at the Institute for Information Industry, Taiwan. They provided a large number of various bad weather scenario sequences which were collected from the road side units, except for the haze scenario. Figure 16 shows the glare scene captured from the sequences collected from Keelung, Taiwan. The contrast was affected by the glare, and Figure 17 shows the corrected result based on our proposed method; Figure 18 is the original image frame of the haze scene condition downloaded from the Yangmingshan National Park’s website [18], and Figure 19 shows the corrected result through our proposed method. The visibilities of the corrected images were apparently increased.

**Figure 20:**
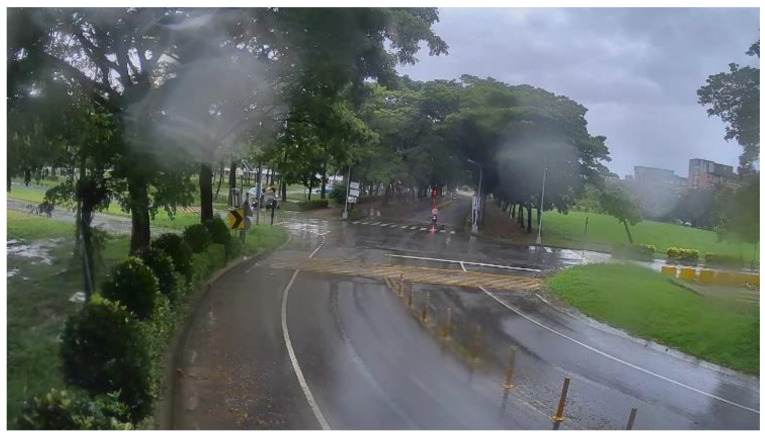
Rainy scene: original image.

**Figure 21:**
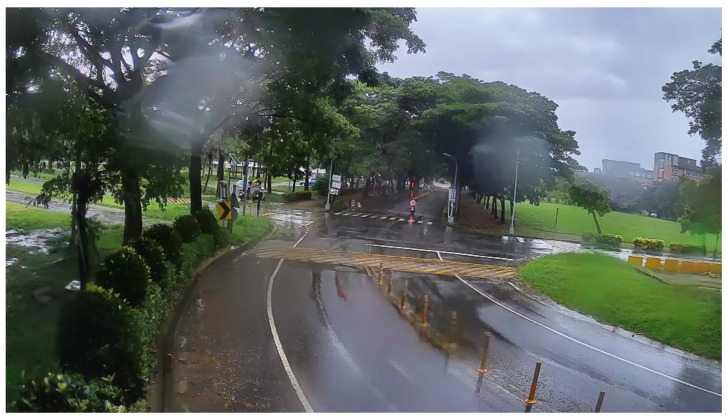
Rainy scene: correction result.

Figure 20 shows the original rainy scene frame captured from the sequences collected from Kaohsiung, Taiwan. Some raindrops adhered to the camera lens and caused distortion. Figure 21 shows the corrected result based on our proposed method.

### 3.2. Performance Evaluation

We applied our system to surveillance videos and compared the detection performance between the YOLOv3 [19] model trained on the same training dataset. The results indicated that our proposed method effectively increased the detection accuracy and was able to reach the real-time requirements under extreme weather conditions.

**Figure 22:**
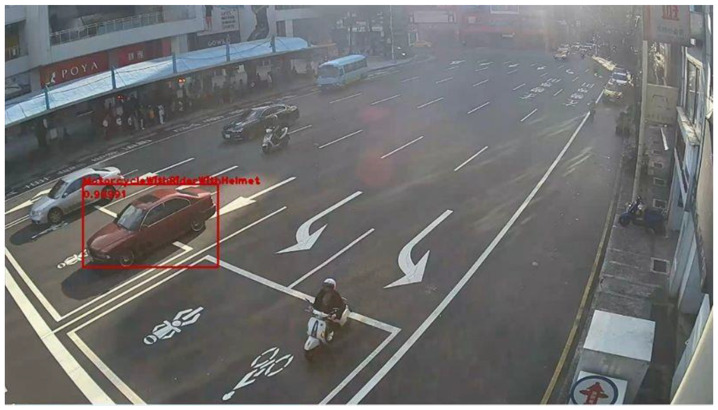
Low-contrast scene image frame detected by YOLOv3.

**Figure 23:**
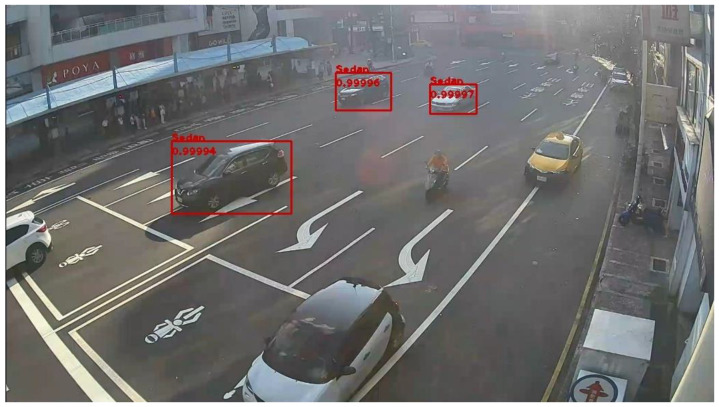
Low-contrast scene image frame detected by YOLOv3.

**Figure 24:**
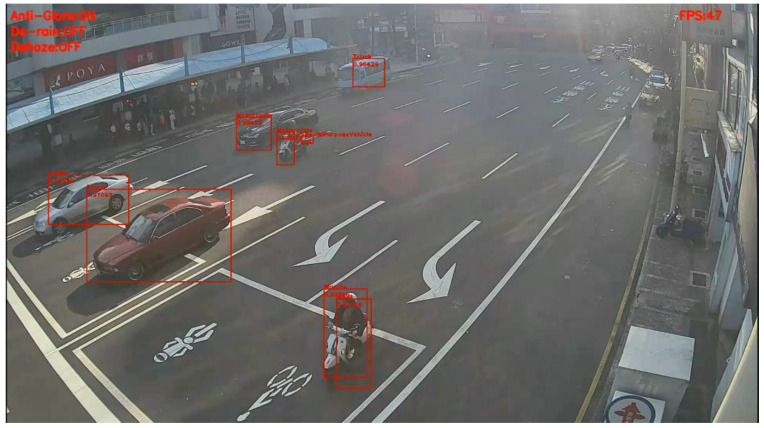
Low-contrast scene image frame detected by our proposed system.

**Figure 25:**
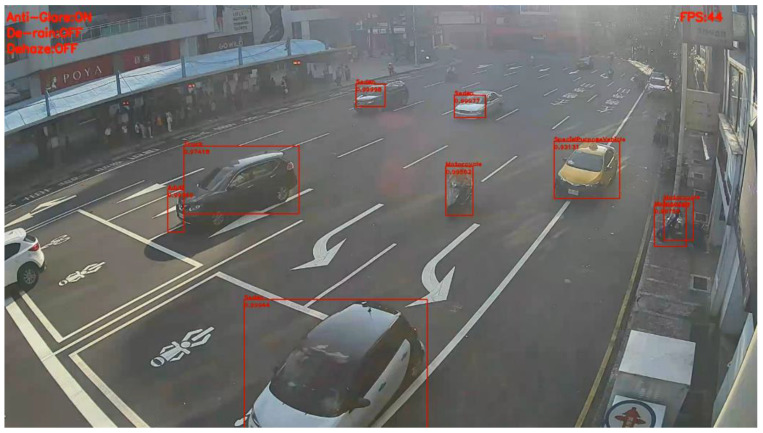
Low-contrast scene image frame detected by our proposed system.

Figure 22 and Figure 23 present the detection results of YOLOv3 applied on the low-contrast scene image frame. Figure 24 and Figure 25 show the detection results of our proposed system. The results showed that, under this condition, YOLOv3 could only detect a few vehicles; however, with our proposed system, the number of detected vehicles was significantly increased.

**Figure 26:**
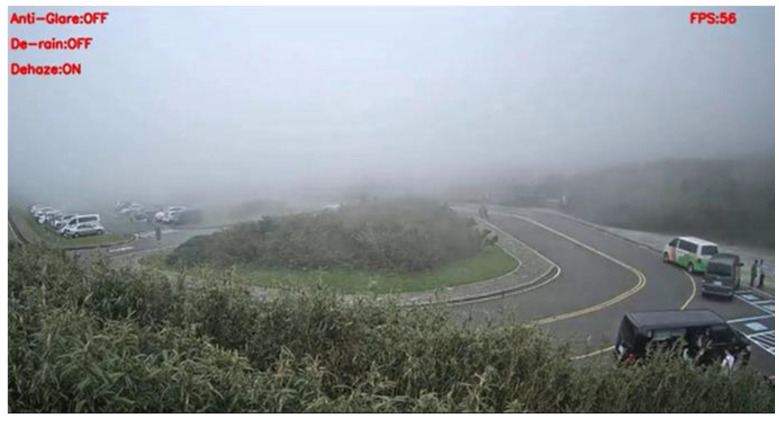
Haze scene image frame detected by YOLOv3.

**Figure 27:**
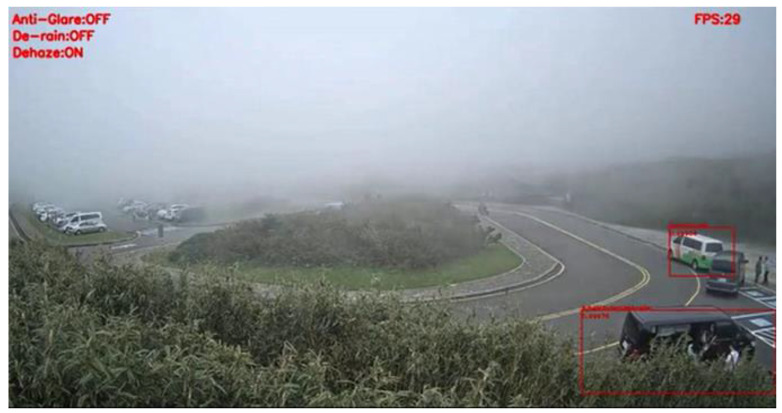
Haze scene image frame detected by our proposed system.

**Figure 28:**
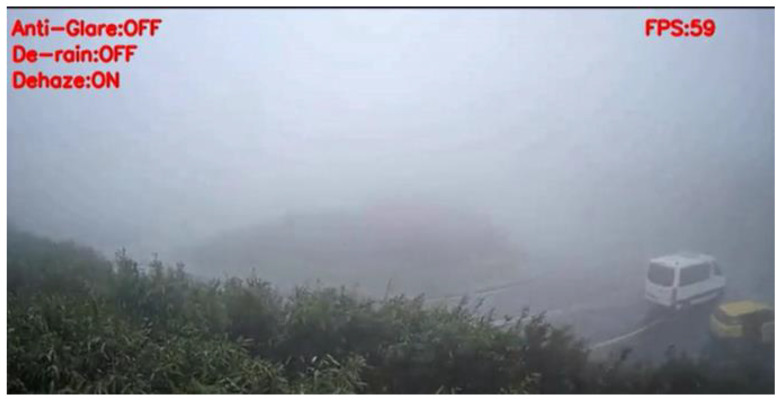
Haze scene image frame detected by YOLOv3.

**Figure 29:**
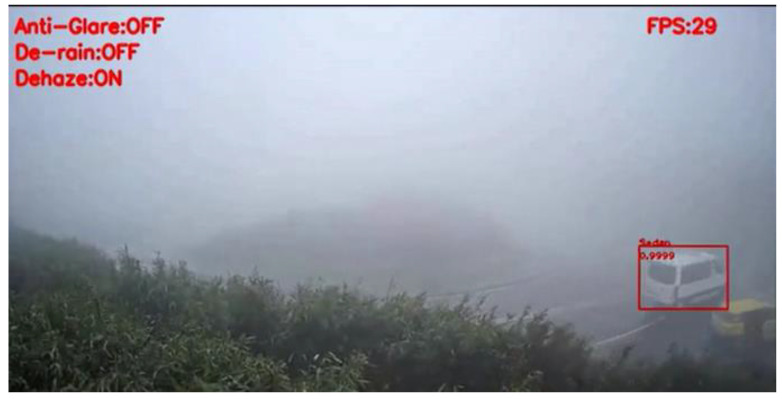
Haze scene image frame detected by our proposed system.

Figure 26 shows the detection result of YOLOv3 applied to a haze scene image frame, and Figure 27 shows the detection result of our proposed system. We can easily observe that YOLOv3 could not detect any vehicle in the image frame; in contrast, our proposed method could detect the black and white vans in the image frame. Under the same scene condition, the detection result of YOLOv3 did not detect any vehicle; in contrast, our proposed system detected a white van and marked it properly, and the details are shown in Figure 28 and Figure 29.

**Figure 30:**
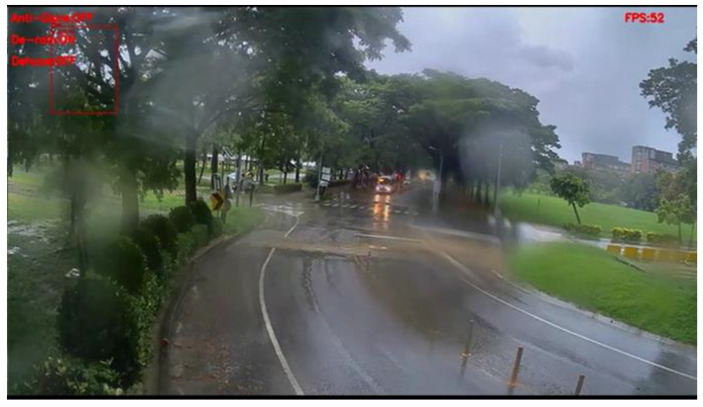
Rainy scene image frame detected by YOLOv3.

**Figure 31:**
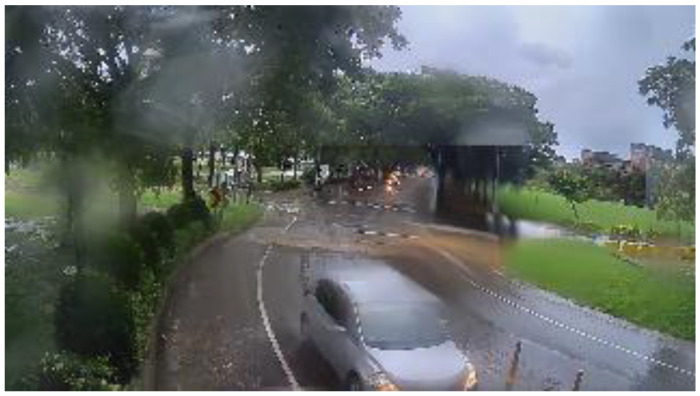
Rainy scene image frame detected by YOLOv3.

**Figure 32:**
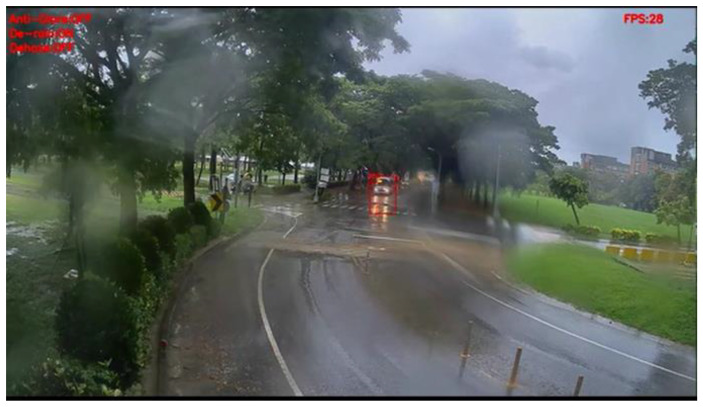
Rainy scene image frame detected by our proposed system.

**Figure 33:**
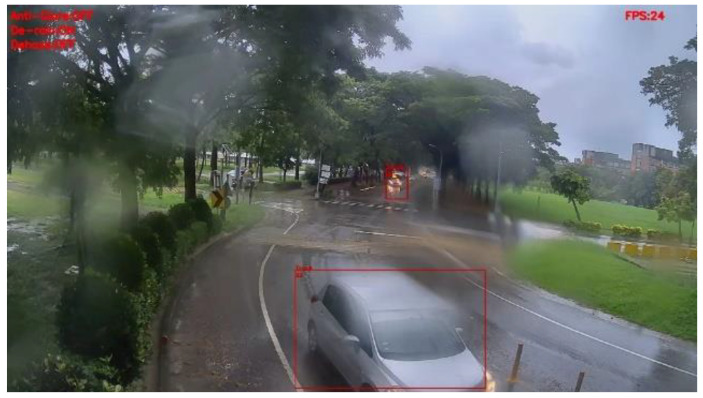
Rainy scene image frame detected by our proposed system.

Figure 30 and Figure 31 show the detection results of YOLOv3 applied to the rainy scene image frame. Figure 32 and Figure 33 show the detection result of our proposed system. As the figure shows, after the visibility correction, the vehicles far away and those covered by raindrops were able to be detected.

In terms of operation time, we tested our proposed system on three different high-definition (HD) sequences for each extreme weather condition. Glare and rainy testing sequences were downloaded from the surveillance system database, and haze testing sequences were downloaded from the Yangmingshan National Park’s website [29]. Table 3 shows the testing results; most of the sequences could reach 30 fps and satisfied the real-time application requirements. Table 4 shows the comparative detection results of applying our proposed method or not on object detection using YOLOv3 [19] with the same pre-trained model.

### 3.3. Comparative Results and Discussion

Through our proposed visibility complementation module, the visibility problem in various bad weather conditions was compensated; as Table 4 shows, under glare scene conditions, the recall rate increased from 85.22% to 89.82%, and the false classification ratio decreased from 14.71% to 9.97%. Furthermore, the recall rate increased over 20% under rainy scene conditions. Under the haze condition, our proposed method could significantly improve the detection ability of traffic objects more than seven-fold; the amount of detected objects increased from 80 to 602. Although the computational time under our testing sequences was able to reach the real-time requirements, it might decrease under high-resolution or complex raindrop conditions, as depicted in Table 3. The computational costs of the image contrast take a great deal of time for high-resolution image frames, and the large number of raindrops under complex raindrop conditions will cause iterative inference through the deep learning model and cost more computational time. The limited effectiveness was caused by the additional noise created during the generation of the non-noise scene; such additional noise affects the clear partitions in the original image frame and results in some bulk false detections and classifications. The background and vehicles were confused by the deep learning model in the generated scene image. As a result, a new complementary method for such haze scene conditions, which is able to preserve the recognition ability of the pre-trained deep learning model, is necessary for vehicle detection system applications in real-world traffic situations.

## 4. Conclusions

The adoption of deep learning techniques for the development of intelligent traffic applications is a main trend in recent research studies, such as vehicle detection and recognition. Although the existing methods perform sufficiently well under general scenarios, the effects under various bad weather conditions are not ideal. As a result, the method for complementing these effects under such scenarios is important. This study proposed a vehicle detection system, including a visibility complementation module, which is able to detect and recognize vehicles under various bad weather conditions without retraining models and is useful for the development of intelligent traffic systems. The improvements in detection and recognition for most of the various bad weather scene conditions were quite obvious from our experiment. Our proposed system was able to increase the accuracy not only by nearly 5% under low-contrast scene condition but also by 50% under rainy scene conditions, which satisfied the real-time requirement. For some specific cases, such as haze conditions, more effort is needed to develop a more efficient method. In future, we will devote more time and energy to the creation of a better correction method which is general and adaptive enough to solve the great majority of bad weather conditions without mass predefined conditions.

## Figures and Tables

**Table 1:** Table of used acronyms.

Acronyms.	Description
$Image_{c}$	The contrast value of each pixel position in the input image.
$\mu_{c}$	The mean value of the input image contrast result.
$T_{c}$	The accepted region of the mean contrast value for a determined low-contrast image frame.
$T_{v}$	The threshold for the determination of a high V value in the Hue-Saturation-Value (HSV) color space.
$R_{high\_v}$	The ratio of high V value pixels to the entire number of pixels in the image.
$T_{high\_v}$	The threshold of the ratio for the determination of low-contrast image frames.
$T_{component}$	The threshold of the number of components for the determination of adherent raindrop conditions.
K	User-defined value of the size of the confirmation reference frame.

**Table 2:** Parameters applied on different scenarios.

Scene Condition	Sequence	Frame Amount (Frames)	Avg. Variance	Ratio of Pixel Amount	Avg. Component Amount
Glare	Glare1	1425	**11,035.14**	3.1874%	1.5
	Glare2	1840	**10,106.54**	1.8145%	2
	Glare3	1780	**9898.337**	1.9105%	2.5
Haze	Haze1	1276	2151.5	**91.4775%**	1
	Haze2	135	2175.344	**88.8172%**	1.5
	Haze3	1276	943.6111	85.57%	2
Sunny	Sunny1	1780	7855.636	8.1367%	1.5
	Sunny2	900	14,106.04	14.9475%	1
Rainy	Rainy1	900	3871.039	31.5765%	**4**
	Rainy2	900	4926.419	32.217%	**4**
	Rainy3	900	4570.564	27.8319%	**5**

**Table 3:** Computation time of the system applied to different weather conditions.

Weather Condition	Low Contrast	Adherent Raindrops	Sequence Name	Frame Amount (Frames)	Total Time (Seconds)	Frames per Second
Glare	☑	⊠	20191002_072701.avi	1779	61.25	29.06
	☑	⊠	20191002_073357.avi	1779	60.56	29.41
	☑	⊠	20191002_073456.avi	1779	59.46	30.30
Haze	☑	⊠	media7.mp4	425	12.97	32.75
	☑	⊠	media8.mp4	425	13.00	32.69
	☑	⊠	media12.mp4	353	10.89	32.40
Rainy	⊠	☑	20190813_083935.avi	899	29.15	31.05
	⊠	☑	20190813_084746.avi	899	29.19	30.79
	⊠	☑	20190813_090641.avi	899	29.40	30.57

**Table 4:** Detection performance comparison between the non-corrected image and corrected image.

Scene		Bounding Box Amount	Positive		False Detection		False Classification	
Glare	Without proposed	5702	4859	(85.22%)	4	(0.07%)	839	(14.71%)
	With proposed	8093	**7269**	**(89.82%)**	17	(0.21%)	**807**	**(9.97%)**
Haze	Without proposed	97	80	(82.47%)	10	(10.31%)	7	(7.22%)
	With proposed	760	**602**	(79.21%)	102	(13.41%)	65	(8.55%)
Rainy	Without proposed	650	435	(66.92%)	172	(26.46%)	43	(6.62%)
	With proposed	998	**871**	**(87.27%)**	**52**	**(5.21%)**	75	(7.52%)
